# Promoting quality through measurement of performance and response: prevention success stories.

**DOI:** 10.3201/eid0702.010231

**Published:** 2001

**Authors:** C Richards, T G Emori, G Peavy, R Gaynes

**Affiliations:** Centers for Disease Control and Prevention, Atlanta, Georgia 30333, USA. cir6@cdc.gov

## Abstract

Successful efforts to prevent health-care acquired infections occur daily in U.S. hospitals. However, few of these "success stories" are presented in the medical literature or discussed at professional meetings. Key components of successful prevention efforts include multidisciplinary teams, appropriate educational interventions, and data dissemination to clinical staff.


### Promoting Quality Through Measurement of Performance and Response: Prevention Success Stories

**Chesley Richards, T. Grace Emori, Gloria Peavy, and Robert Gaynes**
Centers for Disease Control and Prevention, Atlanta, Georgia, USA

Successful efforts to prevent health-care acquired infections occur daily in U.S. hospitals. However, few of these “success stories” are presented in the medical literature or discussed at professional meetings. Key components of successful prevention efforts include multidisciplinary teams, appropriate educational interventions, and data dissemination to clinical staff.

In the past two decades in the United States, demands from patients, insurance companies, managed-care organizations, employers, providers, and policy makers for improved health care have increased dramatically (1). An essential component of quality improvement efforts is performance measurement, the quantification of processes and outcomes by using one or more dimensions of performance (2). Such data can be used for accountability, research, or improvement (3). An important part of the improvement perspective is sharing success stories or “best practices.” We describe key improvement concepts of performance measurement from individual hospitals and selected hospitals in the National Nosocomial Infections Surveillance (NNIS) system.

### Success Stories from Individual Hospitals

### Improving Central-Line Care in Neonates

In 1995, the neonatal intensive care unit (ICU) at Allegheny General Hospital in Pittsburgh, Pennsylvania, underwent substantial expansion. Subsequently, the ICU experienced a 40% increase in very low birth weight (<1,000 g) babies, which resulted in increased overall use of central lines (4). Although the rate of bloodstream infections remained stable during 1995 and 1996, the total number of such infections increased. Concerned neonatal ICU staff formed a multidisciplinary team to develop interventions to prevent them. The team focused on improving procedures for central-line dressings. At the time, central-line sites were covered with gauze and a transparent dressing. The dressing was routinely changed three times each week, which required central-line manipulation. Less frequent changes were not performed because nurses could not see the central-line site, except during dressing changes. The team recommended discontinuing use of gauze over the central-line insertion site but continuing use of the transparent dressings. The team also developed standard protocols for inserting and caring for central lines. Inservice education was provided to nurses and house staff on central-line management. As a result, both the total number and rate of central-line associated bloodstream infections significantly declined in 1997 (Figure 1).

Address for correspondence: Chesley Richards Jr., Division of Healthcare Quality Promotion, NCID, CDC, Mailstop A07, 1600 Clifton Road, Atlanta, GA 30333, USA; fax: 404-639-6483; e-mail: cir6@cdc.gov

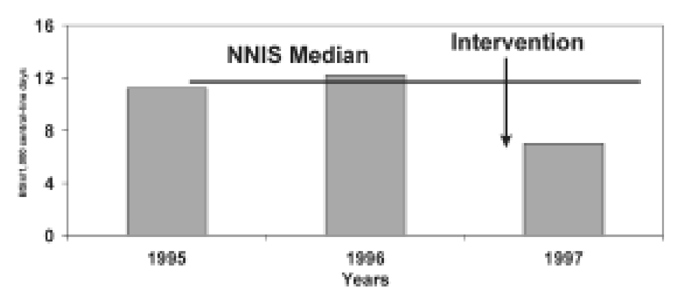

Figure 1. Rates of bloodstream infections (BSIs) associated with central lines in neonatal ICU, Allegheny General Hospital.

### Reducing Use of Urinary Catheters

The Hospital of St. Raphael in New Haven, Connecticut, reported joining NNIS in 1992 (5). Infection control professionals performed infection surveillance in the surgical, medical, and coronary ICUs. During 1992 and 1993, the catheter-associated urinary tract infection rate in all three ICUs was well above median rates for NNIS hospitals. After reviewing urinary tract infections and use of urinary catheters in ICUs, infection control staff identified prolonged use of urinary catheters (mean = 21 days) as the chief risk factor for infection. Although educational sessions to reemphasize existing prevention guidelines for catheter care were conducted with nursing staff, no changes in infection rate were observed. In 1995, a multidisciplinary team was formed to address the system of care for patients with urinary catheters. The team included medical directors, patient-care managers, clinical nurse specialists, physicians, microbiologists, and infection control and quality assurance staff. The team developed a guideline for using urinary catheters, a protocol for removing catheters without a physician’s order, and a protocol requiring urinalyses when urine cultures were ordered. The protocol for removing a catheter required approval by the Connecticut Division of Healthcare Regulation, the hospital medical board, and the hospital's critical care and infection control committees. Nurses, house staff, and attending physicians were extensively educated about the new protocol. Compliance was high for both the physicians' urinalysis protocol (93%) and the nurses' catheter removal protocol (88%). After these protocols were implemented, urinary tract infection rates in all three ICUs decreased and the length of urinary catheter use was shortened.

### Ward-Specific Dissemination of Data

In the Veterans Affairs (VA) Medical Center in Pittsburgh, Pennsylvania, general medical-surgical, non-ICU patients were perceived by clinical staff to have high rates of urinary tract infection. Although there was no national benchmark for comparison, infection control staff used NNIS definitions and data collection methods to calculate ward-specific rates. The major intervention was to disseminate these ward-specific rates to nursing staff. No other changes to policies or protocols were made, and no new products were introduced during this period. After ward-specific feedback was begun, the rate of infections underwent a dramatic and sustained reduction (50%), which saved an estimated $400,000 per year. The authors felt that dissemination of ward-specific rates of urinary tract infection stimulated nurses to improve compliance with prevention guidelines (Figure 2) (6).

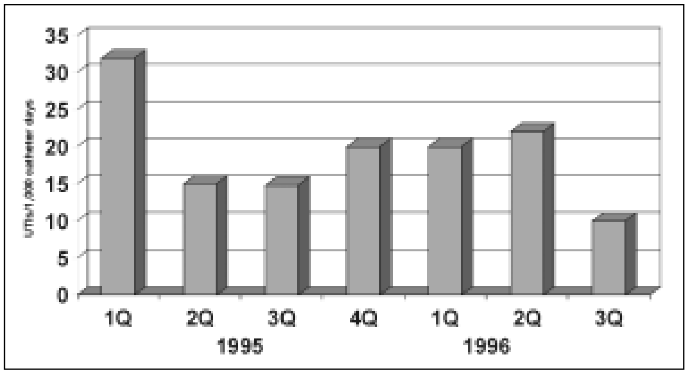

Figure 2. Rates of urinary tract infections (UTIs) in general medical/surgical patients, Pittsburgh VA Medical Center.

### Synthesis of Individual Success Stories

Several key improvement concepts are illustrated by these success stories. Improvements should be determined by local health-care facility needs and involve staff. Comparison to national benchmarks is important for building credibility among clinical staff and allowing facilities to focus attention and resources but does not preclude improvement efforts when external benchmarks are not available or infection rates are relatively low. Since initial improvement efforts may not always succeed, commitment to improvement is vital. Finally, disseminating data back to clinical staff is a simple yet powerful tool for improvement.

### Success Stories from Selected Hospitals in NNIS

### Background and Method

NNIS is the oldest and largest surveillance system for hospital-acquired infections. An important reason for its success has been feedback of data to participating institutions (7). To better understand how surveillance data were used and how institutions worked to reduce infections, NNIS program staff conducted a telephone survey of infection control professionals at NNIS hospitals that had reported reductions in infection rates in ICUs. Specific questions included how their interventions were developed, what types of activities occurred, and how feedback was performed.

### Results

Infection control professionals at 15 (94%) of 16 hospitals responded to the survey. Reductions were reported for ventilator-associated pneumonia (7/15), bloodstream infections (5/15), and urinary tract infections (3/15). While the specific interventions varied at each hospital, the three features common to all 15 institutions were 1) use of multidisciplinary teams, 2) tailored educational interventions directed to clinical staff, and 3) feedback to clinical staff of facility infection rates.

### Multidisciplinary Teams

The primary function of multidisciplinary teams was to build consensus that a problem existed, disseminate information about the infection and any planned interventions to their colleagues, and assist infection control professionals with investigations and prevention. All teams included infection control professionals. Almost all (14/15) teams had physician representation, including a hospital epidemiologist, infectious disease and critical care specialists, and where appropriate other subspecialists (e.g., urologist, pulmonologist). Nursing professionals were present on all teams (15/15) and included critical care nurses and administrative nurses. Most teams (13/15) also included other professionals, such as respiratory therapists, pharmacists, microbiologists, and dieticians.

### Education

Once a particular intervention was identified, educational sessions were used to introduce it and provide training. These educational activities included training for nurses and other ICU staff, multidisciplinary ICU rounds, self-paced educational modules, and for physicians, grand rounds and teaching lectures. In all 15 hospitals, the target audience for interventions included nurses, especially those who were providing direct patient care in the ICU. Less often the interventions included activities directed at physicians (4/15) or respiratory therapists (2/15). Infection control professionals organized and delivered most educational material.

### Data Dissemination

After interventions were reduced, all hospitals disseminated data to their staff on the impact of the interventions on nosocomial infection rates. Data included comparison of hospital infection rates to NNIS benchmarks, intrahospital rates over time, and compliance with interventions. Respondents thought that feedback was most effective when directed at ICU staff and least effective when provided to medical and nursing staff hospitalwide. Data dissemination usually occurred through reports to the ICU staff and infection control committee. Several hospitals (5/15) reported that posting infection rates and protocol compliance as charts or posters in ICU was especially effective.

### Cycle for Success

The reports in this article represent a small yet important collection of efforts directed at preventing infection. While the specific interventions varied, the process in each hospital was strikingly similar. The cycle for success started, for most facilities, with comparisons to external benchmarks. Multidisciplinary teams with diverse representation were formed and identified the "whys" and "whats" for the infections of interest. Such teams also helped formulate the interventions. Education, usually through training sessions with clinical staff, was crucial in introducing change. Feedback of comparative data to staff provided motivation and reinforcement. Comparison to external benchmarks also allowed staff to gauge their success as compared with other institutions. Finally, collaboration across organizational boundaries was a critical element of success (8). These reports effectively demonstrate how collaboration among physicians, nurses, and other professionals was the driving force for these improvement efforts.

As noted by Berwick, reports of performance improvement are desperately needed to guide quality improvement efforts (9). Real-time and real-life improvement reports can provide insights into how health-care quality can be improved. This report is an example of how both individual and aggregate results can inform the improvement process. The Institute of Healthcare Improvement's Breakthrough Series is another example of how improvement success stories are shared with a broader audience (10). However, much important learning for improvement also occurs in community hospitals, ambulatory care centers, long-term care facilities, or physician's offices. Too often, these experiences are not shared. Efforts to increase "harvesting knowledge from improvement" in these settings are needed (9). These efforts should include more success stories, outline epidemiologic approaches to understanding and describing best practices, and increase the use of analysis of root causes.

Dr. Richards is a medical officer with CDC's Division of Healthcare Quality Promotion, formerly the Hospital Infections Program. Dr. Richards' research interests include the epidemiology of health-care acquired infections, antibiotic use in the elderly, and infection control in hospitals and long-term care facilities.

### References

1. Bodenheimer T. The American health care system: The movement for improvement and quality in health care. N Engl J Med 1999;340:488-92.
2. Joint Commission on Accreditation of Healthcare Organizations (JCAHO). The measurement mandate. Oak Brook Terrace (IL): JCAHO; 1993.
3. Solberg LI, Mosser G, McDonald S. The three faces of performance measurement: improvement, accountability, and research. Joint Commission Journal of Quality Improvement 1997;23:135-47.
4. Bishop-Kurylo D. The clinical experience of continuous quality improvement in the neonatal intensive care unit. Journal of Perinatology and Neonatology Nursing 1998;12:51-7.
5. Dumigan D, Kohan CA, Reed CR, Jekel JF, Fikrig MK. Utilizing National Nosocomial Infection Surveillance system data to improve urinary tract infection rates in three intensive care units. Clinical Performance and Quality Improvement in Health Care 1998;6:172-8.
6. Goetz Am, Kedzuf S, Wagener M, Muder RR. Feedback to nursing staff as an intervention to reduce catheter-associated urinary tract infections. Am J Infect Control 1999;27:402-4.
7. Gaynes RP, Solomon S. Improving hospital-acquired infection rates: the CDC's experience. Journal of Quality Improvement 1996;22:457-7.
8. Plsek PE. Collaborating across organizational boundaries to improve the quality of care. Am J Infect Control 1997;25:85-95.
9. Berwick DM. Harvesting knowledge from improvement. JAMA 1996;275:877-8.
10. Kilo C. Improving care through collaboration. Pediatrics 1999;103:384-93.

## References

[PDF_00252] Berwick D. M. (1996). Harvesting knowledge from improvement.. JAMA.

[PDF_00236] Bishop-Kurylo D. (1998). The clinical experience of continuous quality improvement in the neonatal intensive care unit.. J Perinat Neonatal Nurs.

[PDF_00227] Bodenheimer T. (1999). The American health care system--the movement for improved quality in health care.. N Engl J Med.

[PDF_00239] Dumigan D. G., Kohan C. A., Reed C. R., Jekel J. F., Fikrig M. K. (1998). Utilizing national nosocomial infection surveillance system data to improve urinary tract infection rates in three intensive-care units.. Clin Perform Qual Health Care.

[PDF_00247] Gaynes R. P., Solomon S. (1996). Improving hospital-acquired infection rates: the CDC experience.. Jt Comm J Qual Improv.

[PDF_00244] Goetz A. M., Kedzuf S., Wagener M., Muder R. R. (1999). Feedback to nursing staff as an intervention to reduce catheter-associated urinary tract infections.. Am J Infect Control.

[PDF_00254] Kilo C. M. (1999). Improving care through collaboration.. Pediatrics.

[PDF_00250] Plsek P. E. (1997). Collaborating across organizational boundaries to improve the quality of care.. Am J Infect Control.

[PDF_00233] Solberg L. I., Mosser G., McDonald S. (1997). The three faces of performance measurement: improvement, accountability, and research.. Jt Comm J Qual Improv.

